# Whole exome sequencing identified two novel mutations of *ACD* in Chinese patients with idiopathic pulmonary fibrosis

**DOI:** 10.3389/fcell.2026.1765277

**Published:** 2026-02-25

**Authors:** Gao-Hui Cao, Hui Yang, Qian Wang, Hong Luo, Liang-Liang Fan, Lv Liu

**Affiliations:** 1 Department of Pulmonary and Critical Care Medicine, the Second Xiangya Hospital, School of Life Science, Central South University, Changsha, China; 2 Research Unit of Respiratory Disease, Hunan Diagnosis and Treatment Center of Respiratory Disease, the Second Xiangya Hospital, Central South University, Changsha, China

**Keywords:** ACD mutation, idiopathic pulmonary fibrosis, shelterin complex, telomere, TPP1

## Abstract

Idiopathic Pulmonary Fibrosis (IPF) is a progressive, age-related, and distinct form of fibrosing interstitial pneumonia with an unknown etiology. Previous studies have indicated that mutations in the *ACD Shelterin Complex Subunit and Telomerase Recruitment Factor* (*ACD*) gene are associated with the development of IPF. This study aims to investigate *ACD* mutations in Chinese patients with interstitial lung diseases (ILDs). A total of 124 ILD patients were enrolled in this study. Whole exome sequencing and Sanger sequencing were performed to identify genetic variants in these individuals. Mutant plasmids were constructed and transfected into the A549 cell line to conduct *in vitro* functional assays. Among the 124 patients, two novel *ACD* mutations (c.884G>A/p.S295N and c.1074C>G/p.H358Q) were identified in two Chinese families with a history of IPF. Functional analyses revealed that both mutations compromise the stability of the TPP1 protein, leading to reduced TPP1 expression. This downregulation subsequently decreases DKC1 expression, ultimately resulting in telomere shortening and contributing to IPF pathogenesis. To the best of our knowledge, this study represents the first report of *ACD* mutations in an Asian population with IPF. Our findings broaden the mutation and population spectrum of ACD deficiency.

## 1 Introduction

Idiopathic Pulmonary Fibrosis (IPF) is a progressive, age-associated, and distinct form of fibrosing interstitial pneumonia of unknown cause (Maher, 2024). Its diagnosis is defined by radiological and/or histopathological findings that demonstrate a Usual Interstitial Pneumonia (UIP) pattern (Chelala et al., 2025). A central feature of disease pathogenesis is recurrent injury to alveolar epithelial cells, which drives aberrant fibroblast proliferation and activation, resulting in exaggerated deposition of extracellular matrix (Singh et al., 2025). This pathological cascade results in the progressive disruption of normal lung architecture, with functional alveoli being irreversibly replaced by fibrotic scar tissue (Singh et al., 2025). Clinically, this manifests as a steady deterioration in lung function, characterized by progressive dyspnea, a persistent non-productive cough, and eventual respiratory failure. The median survival post-diagnosis is only 3–5 years, underscoring the grave prognosis of this condition (Luo et al., 2025).

Emerging evidence indicates that IPF arises from a complex interplay between environmental exposures and genetic predisposition. Genetic factors are estimated to account for approximately 5%–20% of IPF cases (Newton, 2023). Notably, mutations in telomere-related genes have been linked to accelerated telomere shortening in type II alveolar epithelial cells, promoting cellular senescence (Fernandez and Kropski, 2025). These senescent cells exhibit impaired regenerative capacity and reduced ability to proliferate and differentiate following lung injury. As a result, tissue repair mechanisms shift toward fibroblast-mediated pathways, characterized by fibroblast migration, proliferation, and differentiation (Singh et al., 2025). During this process, fibroblasts accumulate at sites of injury to form fibroblast foci, leading to excessive deposition of extracellular matrix and progressive alveolar fibrosis, ultimately culminating in the development of IPF(Singh et al., 2025). To date, approximately 20 genes involved in telomere maintenance, including *Telomerase Reverse Transcriptase*, *Dyskerin Pseudouridine Synthase 1* (*DKC1*), and *Regulator of Telomere Elongation Helicase 1*, have been identified as pathogenic contributors to IPF(Fernandez and Kropski, 2025).

The human *ACD* gene (NM_001082486.2), located at chromosome 16q22.1, comprises 12 exons spanning approximately 3 kilobases (kb) and encodes the ACD Shelterin Complex Subunit and Telomerase Recruitment Factor, also named TPP1 protein, a component of the shelterin complex and a key recruiter of telomerase (Else et al., 2007). Previous studies have established its essential role in telomere biology. As one of the six core subunits of the shelterin complex, TPP1 is critical for maintaining telomere length and protecting chromosomal ends. It facilitates complex assembly and stabilization through interactions with other shelterin proteins and plays a central role in regulating telomerase recruitment to telomeres (Hu et al., 2017; Agrawal et al., 2025). At present, eleven pathogenic germline *ACD* variants have been reported in 15 individuals from 12 unrelated families with telomere biology disorders, and only six mutations of *ACD* have been reported in IPF patients (Bertrand et al., 2024).

In this study, we performed whole exome sequencing and Sanger sequencing to analyze 124 patients diagnosed with interstitial lung diseases (ILDs). Two novel *ACD* gene mutations (c.884G>A/p.S295N and c.1074C>G/p.H358Q) were identified in two Chinese families affected by IPF. Telomere length assessment and *in vitro* functional analyses demonstrated that both mutations compromise the stability of the TPP1 protein, leading to reduced levels of TPP1 protein. This reduction was associated with decreased expression of DKC1, a key telomere-related gene, and concomitant telomere shortening.

## 2 Materials and methods

### 2.1 Subjects

A total of 124 unrelated patients diagnosed with ILDs at the Second Xiangya Hospital were enrolled in the study (Supplementary Table S1). In the families reported herein, blood samples were collected from each family, including the health controls and affected individuals. High-resolution computed tomography (CT) was performed to assess the affected individuals.

### 2.2 Whole-exome sequencing and sanger sequencing

Genomic DNA was isolated from peripheral blood lymphocytes of all participants using the DNeasy Blood & Tissue Kit (Qiagen, Cat. No. 69504) following the manufacturer’s instructions. Whole-exome sequencing (WES) of two probands were conducted at BerryGenomics Biotech Company (Beijing, China), as described previously (Wang et al., 2024). The variant filtering workflow was implemented according to the following rigorously defined inclusion and exclusion criteria: (1) exclusion of all non-coding synonymous variants; (2) removal of non-synonymous single-nucleotide polymorphisms (SNPs) and frameshift-inducing insertions/deletions (INDELs) with a minor allele frequency (MAF) exceeding 0.01 in population databases including dbSNP build 144, the 1000 Genomes Project, the NHLBI Exome Sequencing Project Exome Variant Server (ESP6500), the Genome Aggregation Database (gnomAD), and an in-house control cohort comprising 2,500 exomes; (3) retention of SNPs and INDELs predicted to be deleterious by both SIFT (Sorting Intolerant From Tolerant) and MutationTaster; (4) inclusion of variants exhibiting a Combined Annotation Dependent Depletion (CADD) score >10 and a Deep Learning Annotation of Variants (DANN) score >0.9; and (5) experimental validation of familial co-segregation using Sanger sequencing. PCR primers were designed with Primer Premier 5 software (primer sequences available upon request), and bidirectional Sanger sequencing of amplified fragments was performed on an ABI 3100 Genetic Analyzer (Applied Biosystems, Foster City, CA, United State).

### 2.3 Plasmid construction and cell culture

The wild-type *ACD* coding sequence (NM_001082486.2) with a C-terminal Flag tag was cloned into the pEnter vector. Two *ACD* missense variants (c.884G>A, p.S295N and c.1074C>G, p.H358Q) were individually introduced using the Mut Express II Fast Mutagenesis Kit (Vazyme, C214-01). A549 cells, maintained at 37 °C and 5% CO_2_ in Dulbecco’s Modified Eagle Medium supplemented with 10% fetal bovine serum, 50 IU/mL penicillin, 50 μg/mL streptomycin, and glutamine, were transfected with 1 μg of the respective plasmid (wild-type or mutant) using Lipofectamine™ 2000 CD Transfection Reagent (Invitrogen, 12566014).

### 2.4 Western blot analysis

Proteins were extracted from transfected A549 cells using RIPA lysis buffer, and concentrations were determined with the Pierce™ BCA Protein Assay Kit (Thermo Fisher, 23,225). Total protein (30 μg per lane) was separated on 4%–12% Bis-Tris NuPAGE gels (Invitrogen, EC6026BOX) and transferred to PVDF membranes. After blocking, membranes were incubated with primary antibodies against Myc (Abcam, ab206486), DKC1 (Abcam, ab156877), Calnexin (Abcam, ab22595) or GAPDH (Abcam, ab9484), followed by HRP-conjugated secondary antibodies. Protein bands were visualized using a chemiluminescent imaging system (Alpha Innotech). Protein expression levels were quantified using ImageJ software via grayscale densitometry analysis. For each independent experiment, the expression levels of target genes in cells transfected with the wild-type (WT) plasmid were set as the reference (normalized to 1.0), and expression levels in mutant plasmid-transfected cells were calculated relative to this baseline. All experiments were repeated independently at least three times.

### 2.5 Immunofluorescence analysis

Cells were fixed with 4% paraformaldehyde, permeabilized with 0.5% Triton X-100, and blocked before incubation with anti-Myc antibody (Abcam, ab206486). After washing, cells were incubated with Alexa Fluor 488-conjugated secondary antibody (Thermo Fisher, A-11008) and counterstained with DAPI (Thermo Fisher, 62,247). Images were acquired using a Leica SP5 confocal microscope following standard protocols.

### 2.6 Telomere length measurement

The telomere length of cells was detected by real-time PCR using 50 ng of genomic DNA. The sequence of primers is as follows: telomere F: CGG​TTT​GTT​TGG​GTT​TGG​GTT​TGG​GTT​TGG​GTT​TGG​GTT; telomere R: GGC​TTG​CCT​TAC​CCT​TAC​CCT​TAC​CCT​TAC​CCT​TAC​CCT; β-globin F: GCT​TCT​GAC​ACA​ACT​GTG​TTC​ACT​AGC; β-globin R: CAC​CAA​CTT​CAT​CCA​CGT​TCA​CC. Human peripheral blood mononuclear cells telomere length was assessed by real-time PCR using 50 ng of genomic DNA and the Biowing Telomere Detection Kit (Shanghai Biowing Applied Biotechnology), which was pre-calibrated against a reference cohort of 1,500 random peripheral blood samples from Shanghai (Sun et al., 2021). Reactions were performed on a Fast 7,500 Real-Time PCR System (Applied Biosystems), and telomere length was calculated using the 2^(−ΔΔCt)^ method.

### 2.7 Statistical analysis

All data were analyzed with GraphPad Prism 8 and are presented as mean ± SEM from at least three independent experiments. Differences between two groups were assessed by two-tailed Student’s t-test, and multiple comparisons were performed by ANOVA. A p-value <0.05 was considered statistically significant (*p < 0.05, **p < 0.01, ***p < 0.001).

## 3 Results

### 3.1 Clinical and genetic analysis of family 1

**FIGURE 1:**
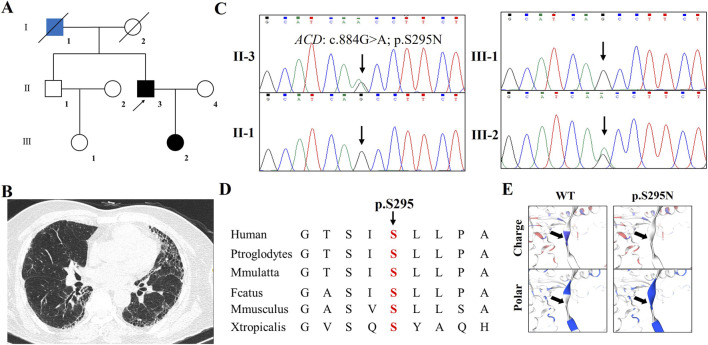
The clinical and genetic analysis of Family 1 **(A)** Pedigree of the family 1 affected with IPF. Family members are identified by generations and numbers. Squares indicate male family members; circles, female members; Blue closed symbols, the affected lung adenocarcinoma individual; black closed symbols, the affected IPF members; open symbols, unaffected members; arrow, proband **(B)** The HRCT of the proband **(C)** Sanger DNA sequencing chromatogram demonstrates the heterozygosity for an *ACD* missense mutation (c.884G>A; p.S295N) in the family **(D)** Alignment of multiple TPP1 protein sequences across species. The S295 affected amino acid locates in the highly conserved amino acid region in different mammals (from Ensembl). Red column shows the S295 site **(E)** The wild type TPP1 (WT) protein structure and the mutant TPP1 (p.S295N) protein structure were predicted by SWISS-MODEL online software. The surface charge and polarity of the WT and mutated TPP1 were predicted.

In Family 1 (Figure 1A), the proband (F1-II-3), a 75 year-old male, presented with a three-month history of cough. Physical examination revealed that the patient’s bilateral thorax was symmetrical without deformity. Percussion of both lungs was clear, and auscultation of breathing was clear. Velcro rales could be heard. HRCT demonstrated Interstitial pneumonia of both lungs, partial wall thinning, UIP type, scattered small nodules in both lungs (Figure 1B). Serological testing for lung cancer biomarkers, respiratory pathogens, a comprehensive tuberculosis panel, and connective tissue disease antibodies all returned negative results. A multidisciplinary team (MDT) consultation involving specialists in pulmonology, radiology, and rheumatology concluded that the patient fulfilled the diagnostic criteria for interstitial pneumonia. Family history indicated that his son (F1-III-2) had a history of hemoptysis and was previously diagnosed with ILD at another institution 3 years ago. His father (F1-I-1) died of lung adenocarcinoma. Following whole-exome sequencing and data filtering using the aforementioned methods, 12 variants were retained (Supplementary Table S2). Among these, the novel *ACD* mutation (c.884G>A; p.S295N) was considered the most likely pathogenic variant responsible for the familial phenotype. Sanger sequencing confirmed co-segregation of this mutation with affected individuals (F1-II-3 and F1-III-2) and its absence in unaffected family members (F1-II-1 and F1-III-1) (Figure 1C), as well as in 200 internal control subjects. The mutation results in a substitution of serine by asparagine at a highly evolutionarily conserved residue within the TPP1 protein (Figure 1D). Structural analysis further revealed that the p.S295N substitution alters the polar and surface charge distribution of the TPP1 protein (Figure 1E).

### 3.2 Clinical and genetic analysis of family 2

**FIGURE 2:**
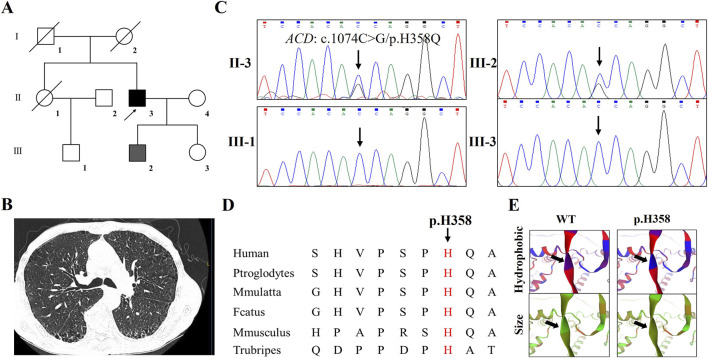
The clinical and genetic analysis of Family 2 **(A)** Pedigree of the family 2 affected with IPF and COPD. Family members are identified by generations and numbers. Squares indicate male family members; circles, female members; grey closed symbols, the affected COPD individual; black closed symbols, the affected IPF member; open symbols, unaffected members; arrow, proband **(B)** The HRCT of the proband **(C)** Sanger DNA sequencing chromatogram demonstrates the heterozygosity for an *ACD* missense mutation (c.1074C>G/p.H358Q) in the family **(D)** Alignment of multiple TPP1 protein sequences across species. The H358 affected amino acid locates in the highly conserved amino acid region in different mammals (from Ensembl). Red column shows the H358 site **(E)** The wild type TPP1 (WT) protein structure and the mutant TPP1 (p.H358Q) protein structure were predicted by SWISS-MODEL online software. The hydrophobic and size of the WT and mutated TPP1 were predicted.

In Family 2 (Figure 2A), the proband (F2-II-3), a 70-year-old male, was admitted to our hospital due to a 1 month history of progressive dyspnea. Physical examination revealed no deformity in the patient’s thorax, symmetrical bilateral respiratory activities, clear sounds on percussion of both lungs, low breathing sounds in both lungs, and no dry or wet rales or pleural friction sounds were heard. HRCT showed a mass in the posterior basal segment of the lower lobe of the left lung, multiple small nodules in both lungs, emphysema, bullae with a little inflammation in both lungs, and interstitial lesions in both lower lungs (Figure 2B). Laboratory tests, including autoantibody screening for connective tissue diseases, lung cancer markers, respiratory pathogen panels, and a comprehensive tuberculosis evaluation, were all negative. An MDT review confirmed the diagnosis of IPF based on clinical, radiological, and histopathological criteria. Family history revealed that his daughter (F2-III-2) had been diagnosed with chronic obstructive pulmonary disease (COPD) 5 years prior. Whole exome sequencing followed by stringent filtering identified 10 candidate variants, including a novel *ACD* mutation (c.1074C>G; p.H358Q) (Supplementary Table S2). Sanger sequencing validated that this variant co-segregated with affected family members (Figure 2C) and was absent in 200 control cohorts. Multiple sequence alignment across species demonstrated that the affected amino acid residue is evolutionarily conserved (Figure 2D). Structural modeling indicated that the p.H358Q mutation affects the hydrophilic properties and electrostatic surface potential of the TPP1 protein (Figure 2E).

### 3.3 Functional studies of two novel mutations

**FIGURE 3:**
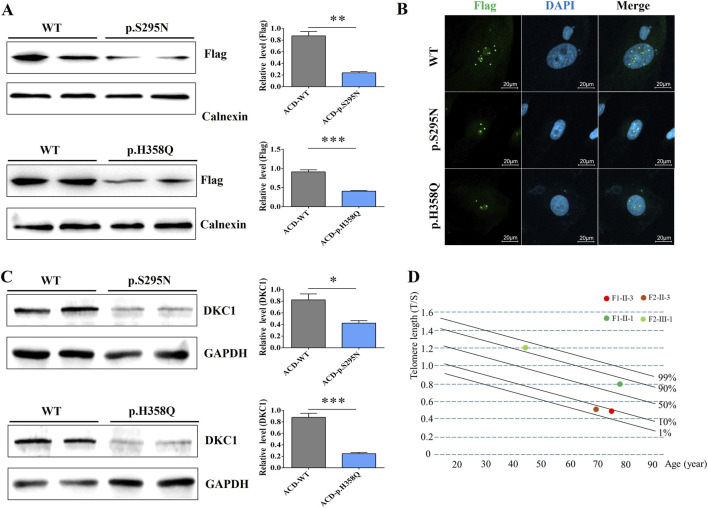
Functional studies of two novel mutations of *ACD* **(A)** Western blot detects the expression of transfected *ACD* (Flag) in the WT and mutated groups **(B)** Immunofluorescence staining showing the subcellular localization of WT and two mutant TPP1 proteins. Flag represents the transfected WT or mutant ACD plasmids, and DAPI represents the cell nucleus **(C)** Western blot analysis of the levels of DKC1 and GAPDH from cells transfected with WT or mutant *ACD* plasmids **(D)** Telomere length of the mutation carriers (F1-II-3 and F2-II-3) and healthy controls (F1-II-1 and F2-III-1).

To investigate the functional consequences of the identified mutations, we constructed plasmids encoding wild-type (WT) and mutant (p.S295N and p.H358Q) ACD proteins and transfected them into the A549 cell line, respectively (Supplementary Figure S1). Western blot analysis revealed a significant reduction in Flag-tagged ACD protein expression in both mutant groups compared to the WT control (Figure 3A). Immunofluorescence staining confirmed diminished nuclear localization of the mutant ACD proteins (Figure 3B). Furthermore, Western blot analysis showed markedly reduced expression levels of DKC1, a key gene involved in telomere maintenance, in cells transfected with either mutated plasmid compared to those cells transfected with WT plasmid (Figure 3C). Telomere length detection of cells showed that the length of telomeres cells transfected with mutated plasmids were shorter than transfected with WT plasmids (Supplementary Figure S2). Telomere length assays in human demonstrated that both mutation carriers exhibited significantly shorter telomeres than healthy controls (Figure 3D). Collectively, these functional studies indicate that both novel *ACD* mutations lead to protein destabilization, impaired expression of telomere-associated genes such as DKC1, and accelerated telomere shortening.

## 4 Discussion

**FIGURE 4:**
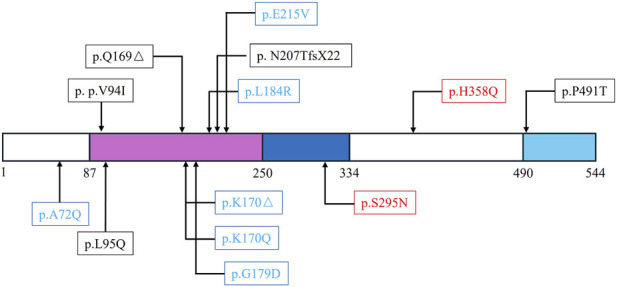
The summary of reported *ACD* mutations. The blue squares indicated patients with IPF. The red square indicated the present study.

Emerging evidence highlights the critical role of telomere biology-related genetic factors in the pathogenesis and progression of IPF(Newton, 2023; Fernandez and Kropski, 2025). Among these genes, *ACD* is involved in telomere length maintenance and end protection, thereby playing a key role in regulating senescence in type II alveolar epithelial cells (Bertrand et al., 2024). In 2019, Thijs W. Hoffman et al. first described three mutations of *ACD* were associated with pulmonary fibrosis in Netherlands IPF population (Hoffman et al., 2019). Recently, Alexis Bertrand et al. detected another two mutations of *ACD* in the French IPF families (Bertrand et al., 2024) (Figure 4). Here, we identified two novel *ACD* mutations (c.884G>A/p.S295N and c.1074C>G/p.H358Q) among 124 Chinese patients with ILDs. Family history analysis revealed that the p.S295N variant was present in individuals diagnosed with IPF and COPD, whereas the p.H358Q variant was associated with IPF. Functional assays demonstrated that both mutations impaired TPP1 protein stability and reduced its nuclear expression. These alterations subsequently downregulated the expression of DKC1, a known IPF-related gene (Gaysinskaya et al., 2020), ultimately leading to telomere shortening and promoting IPF pathogenesis. Our findings align with previous reports linking *ACD* mutations to short telomere syndromes, which can manifest as IPF, dyskeratosis congenita, microcephaly, failure to thrive, speech delay, severe B-cell deficiency with life-threatening infections, enteropathy, and bone marrow hypocellularity (Guo et al., 2014; Tummala et al., 2018; Hoffman et al., 2019; Henslee et al., 2021). According to the ACMG guidelines (Richards et al., 2015), the p.S295N mutation is classified as pathogenic (criteria: PS3 + PM1 + PM2 + PP1 + PP3), and the p.H358Q variant is classified as likely pathogenic (PS3 + PM2 + PP1 + PP3).

Telomere length regulation and chromosome end capping are critical for maintaining genome stability and are primarily mediated by the shelterin and CST complexes (Liu et al., 2022; Cai et al., 2024). The Protection of telomere 1 (POT1)-TPP1, a key subunit of the shelterin complex, binds to the single-stranded telomeric overhang, suppresses the Ataxia Telangiectasia and Rad3-related (ATR)-dependent DNA damage response (DDR) (Gu et al., 2021), and facilitates telomerase recruitment to telomeres to support telomere elongation (Rajavel et al., 2014; Aramburu et al., 2020). Previous studies have demonstrated that loss of TPP1 led to reduced POT1 occupancy at telomeres, impaired telomerase processivity, activation of the ATR-dependent DDR pathway, and p53-mediated cell cycle arrest (Kibe et al., 2017). In this study, two missense mutations (c.884G>A/p.S295N and c.1074C>G/p.H358Q) were found to compromise the stability of the TPP1 protein, resulting in decreased TPP1 expression levels in cell nuclear. This reduction is likely to diminish POT1 association with telomeres, thereby impairing telomerase function and ultimately leading to telomere shortening. Our findings further underscore the essential role of the POT1-TPP1 subcomplex in telomere protection and length maintenance and highlight the significance of telomere-related genes in the pathogenesis of IPF.

Prior to the establishment of the association between the *ACD* gene and IPF, POT1-TPP1 mutations had been implicated in several cancer types, including melanoma, glioma, and chronic lymphocytic leukemia (Aoude et al., 2015). Notably, the p. P507L variant was found to increase the risk of colorectal cancer in Chinese population (Li et al., 2018). Mutations (p.V272M and p.I322F) located within the POT1-binding domain of TPP1 were identified in patients with familial melanoma (Shi et al., 2014; Aoude et al., 2015). The TPP1 protein comprises four distinct domains: an N-terminal domain (NTD, residues 1–86), an oligosaccharide/oligonucleotide-binding fold domain (OBD, residues 87–249), a POT1-binding domain (PBD, residues 250–333), and a C-terminal domain containing a TIN2 interaction region (CTD, residues 334–544) (Aureli et al., 2023). Previously reported *ACD* mutations in IPF patients were localized to the OBD and NTD domains (Hoffman et al., 2019; Bertrand et al., 2024). In this study, the p.S295N mutation was found in the POT1-binding domain; in addition to potentially affecting POT1 levels by compromising TPP1 stability, it may also impair the interaction between TPP1 and POT1, thereby contributing to reduced telomerase activity and telomere shortening. The p.H358Q mutation, located in the C-terminal domain, similarly affected TPP1 stability and ultimately led to telomere shortening. This represents the third report worldwide describing *ACD* mutations in IPF patients, with both mutations being newly identified in the Asian population. Our findings expand the mutation and population spectrum of *ACD* and provide novel insights into the roles of the PBD and CTD domains of TPP1 in telomere maintenance.

Patients harboring *ACD* mutations exhibit marked genetic heterogeneity, particularly with respect to disease manifestations and age of onset. In addition to IPF, a range of other clinical phenotypes including dyskeratosis congenita, bone marrow failure, and severe immunodeficiency have been observed in individuals with *ACD* mutations (Guo et al., 2014; Tummala et al., 2018; Hoffman et al., 2019; Henslee et al., 2021). The affected members from both families in this study likewise demonstrate significant phenotypic variability. For instance, in Family 1, the proband (F1-II-3) was diagnosed with IPF at the age of 75 years, whereas his affected son developed symptoms at 47 years old. Further evaluation revealed that the proband had no history of cigarette smoking, while his son had a 20 year smoking history, which may have contributed to earlier symptom onset and accelerated disease progression (Shi et al., 2025). In addition, in Family 2, the proband’s daughter carried the p.H358Q mutation but present with COPD phenotype. Previous study suggested that telomere-related mutations account for approximately 1% of COPD patients (Hoffman et al., 2018). Our previous studies have also found two patients who carried telomere-related mutations and presented with COPD (Liu et al., 2023; Fan et al., 2025). Certainly, the manifestation of COPD can also be influenced by additional environmental factors (Sese and Annesi-Maesano, 2024). Our study suggested that the COPD phenotype might be affected by telomere-related mutation.

The c.1074C>G/p.H358Q mutation in the *ACD* gene has not been documented in any public databases, including 1,000 Genomes (1000G), ExAC, gnomAD, or ClinVar. The c.884G>A; p.S295N mutation in *ACD* was not observed in the 1000G database but is present in gnomAD with a minor allele frequency (MAF) of 0.000007 (rs760978454). As a late onset pulmonary disease, the IPF usually showed symptoms after the age of 50. This delayed onset may account for the extremely low MAF of this variant in population databases such as gnomAD. As a rare mutation, the mutation (c.884G>A; p.S295N) also exists in patients with dyskeratosis congenita and inborn genetic diseases in ClinVar database. However, no studies have yet reported this mutation in *ACD* among IPF patients.

In summary, through comprehensive analysis of whole exome sequencing data from 124 patients with ILDs, we identified two novel *ACD* gene mutations (c.884G>A/p.S295N and c.1074C>G/p.H358Q) in two Chinese families affected by IPF. Functional studies demonstrated that both mutations impair the stability of the TPP1 protein, resulting in reduced TPP1 expression, which subsequently downregulates DKC1 expression and ultimately leads to telomere shortening and the development of IPF. This study expands the mutation and population spectrum of ACD deficiency and provides new insights into the role of the shelterin complex in IPF and related disorders.

### 4.1 Limitations

Certainly, this study has several limitations. First, lung biopsies were not performed due to the refusal of *ACD* mutation carriers and their relatives to provide frozen tissue samples. If such samples could be acquired and analyzed by single cell transcriptome sequencing (Chu et al., 2022), it may be possible to more precisely elucidate subcellular population dynamics and alterations in key signaling pathways associated with *ACD* mutations during the progression of IPF. Second, there is currently a lack of animal models harboring *ACD* mutations. The development of a knock-in mouse model carrying an *ACD* mutation would represent a valuable approach for investigating the functional role of these mutations in the pathogenesis of IPF, especially for *ACD* gene which have been linked to a spectrum of clinical phenotypes, including pulmonary fibrosis, dyskeratosis congenita, and Høyeraal-Hreidarsson syndrome (Bertrand et al., 2024). Third, the detailed molecular mechanisms by which these two mutations compromise TPP1 protein stability remain unclear. High-resolution techniques, such as cryo-electron microscopy may be an appropriate tool to prove that both mutations disrupted the structure of TPP1 protein and further affected the stability of the TPP1 protein. Finally, the A549 cell line is a lung adenocarcinoma cell line which can be used in mutation functional studies, but it is not quite suitable to conduct IPF signaling pathway research, perhaps primary cultured alveolar type II (AT2) cells would be more suitable.

## Data Availability

The raw data supporting the conclusions of this article will be made available by the authors, without undue reservation.
