# Supplementary material for: Whole exome sequencing identified two novel mutations of ACD in Chinese patients with idiopathic pulmonary fibrosis
Source: Front Cell Dev Biol. 2026 Feb 25;14:1765277. doi: 10.3389/fcell.2026.1765277 (PMC12976011; doi:10.3389/fcell.2026.1765277)
Supplement: Supplementary file 1 [file DataSheet1.docx]

Whole exome sequencing identified two novel mutations of *ACD* in Chinese patients with idiopathic pulmonary fibrosis and chronic obstructive pulmonary disease

Gao-Hui Cao^1#^, Hui Yang^1#^, Qian Wang^1^, Hong Luo^2^, Liang-Liang Fan^1*^, Lv Liu^1,2*^

^1^ Department of Pulmonary and Critical Care Medicine, the Second Xiangya Hospital, School of Life Science, Central South University, Changsha, China.

^2^ Research Unit of Respiratory Disease, Hunan Diagnosis and Treatment Center of Respiratory Disease, the Second Xiangya Hospital, Central South University, Changsha, China.

#contributed equally.

*Corresponding authors

Liang-Liang Fan Ph.D

E-mail: [swfanliangliang@csu.edu.cn](mailto:swfanliangliang@csu.edu.cn)

and

Lv Liu, M.D

E-mail: [docliulv@csu.edu.cn](mailto:docliulv@csu.edu.cn)


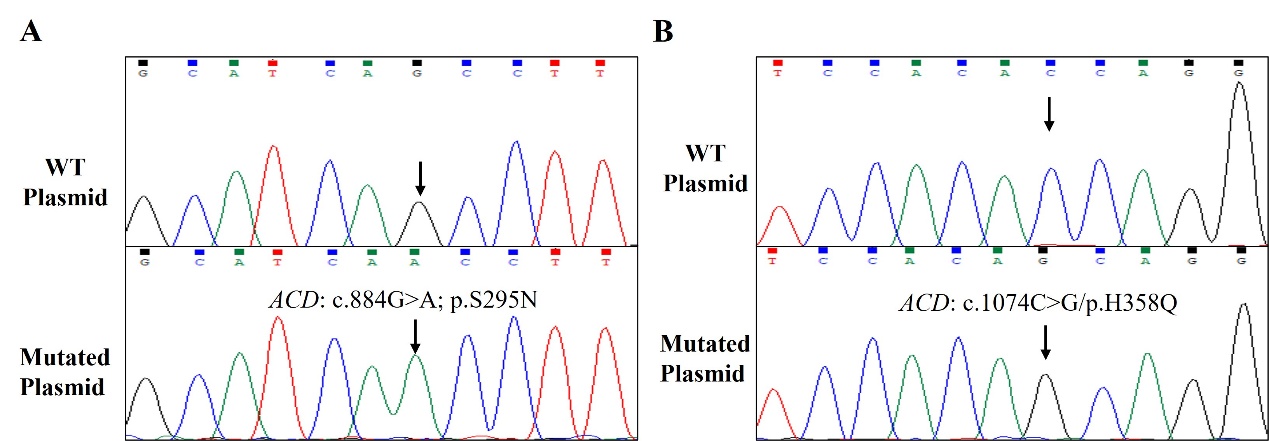


Figure S1. Sanger DNA sequencing demonstrates the *ACD* missense mutations c.884G>A/p.S295N (A) and c.1074C>G/p.H358Q (B) in the mutated plasmids.


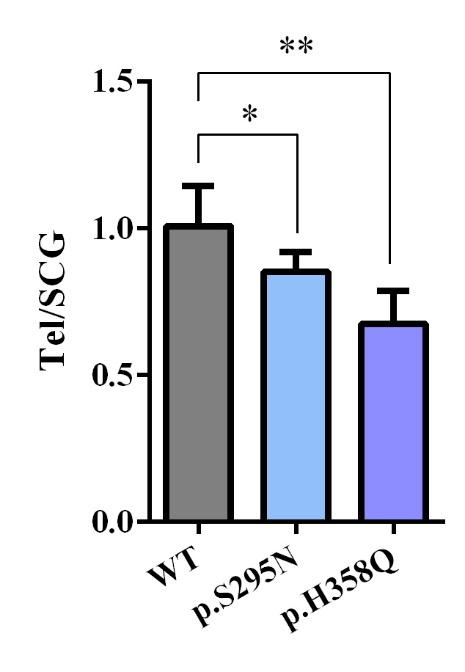


Figure S2. Comparison of relative telomere length. Y-axis (Tel/SCG): represents the ratio of the amplification products of telomeres (Telomere, Tel) to single-copy genes (Single-copy gene, SCG).

Table S1. The clinical characteristics of patients with interstitial lung disease.

| Characteristics | ILD cases (n=124) |
| --- | --- |
| Age | 62.88±11.06 |
| Gender |  |
| Male | 90 (72.58%) |
| Female | 34 (27.42%) |
| Smoking status |  |
| Former/current | 76 (61.29%) |
| Never | 48 (38.71%) |
| Clinical manifestation |  |
| Cough | 112 (90.32) |
| Dyspnea | 95 (76.61%) |
| Velco rales | 88 (70.97%) |
| Gastroesophageal reflux disease | 10 (8.06%) |
| Liver disease | 19 (15.32%) |
| Diabetes | 18 (14.51%) |

Table S2. The mutation list after data filtering in two families.

| F | Chr | POS | RB | AB | Gene | Mutation |
| --- | --- | --- | --- | --- | --- | --- |
| 1 | 2 | 28849919 | G | A | SPDYA | NM_001142634.2: c.920G>A:p.W307X |
|  | 4 | 102905528 | T | A | SLC9B1 | NM_001100874.3: c.1318A>T:p.K440X |
|  | 4 | 102905612 | G | A | SLC9B1 | NM_001100874.3: c.1234C>T:p.R412X |
|  | 7 | 6331075 | G | T | FAM220A | NM_001037163.2: c.80C>A:p.S27X |
|  | 9 | 41906949 | T | A | CNTNAP3B | NM_001201380.3: c.3259A>T:p.N1087Y |
|  | 9 | 41960814 | C | A | CNTNAP3B | NM_001201380.3: c.1835G>T:p.S612I |
|  | 9 | 130369689 | C | T | HMCN2 | XM_017014585.1: c.3688C>T:p.R1230W |
|  | 10 | 3113516 | C | T | PFKP | NM_001323067.2:c.1288C>T:p.Q430X |
|  | 16 | 28723529 | C | T | EIF3C | NM_001037808.2: c.1018C>T:p.R340X |
|  | 16 | 67658308 | C | T | ACD | NM_001082486.2: c.884G>A:p.S295N |
|  | 21 | 10590518 | G | A | TPTE | NM_001290224.2: c.670G>A:p.A224T |
|  | 21 | 10603571 | A | G | TPTE | NM_001290224.2:c.1045A>G:p.T349A |
| 2 | 3 | 31830142 | A | C | OSBPL10 | NM_017784.5: c.627T>G:p.S209R |
|  | 3 | 51704014 | T | C | TEX264 | NM_001243727.3: c.718T>C |
|  | 7 | 101210414 | C | A | PLOD3 | NM_001084.5: c.1531G>T:p.E511X |
|  | 9 | 33750660 | C | T | PRSS3 | XM_011517965.1: c.124C>T:p.P42S |
|  | 10 | 70749961 | C | T | ADAMTS14 | XM_011539306.2: c.2368C>T:p.P790S |
|  | 10 | 97679759 | C | A | AVPI1 | NM_021732.3: c.147G>T:p.Q49H |
|  | 10 | 122428323 | A | T | PLEKHA1 | XM_011540018.1: c.1013A>T:p.H338L |
|  | 16 | 67658118 | G | C | ACD | NM_001082486.2: c.1074C>G:p.H358Q |
|  | 19 | 44176938 | T | G | ZNF226 | NM_001032372.2: c.1676T>G:p.F559C |
|  | 20 | 43513520 | A | G | L3MBTL1 | NM_001377303.1:c.17A>G:p.E6G |

F, Family; Chr, Chromosome; POS, position; RB, reference sequence base; AB, alternative base identified.
